# Replacing the immune system of the brain

**DOI:** 10.7554/eLife.110753

**Published:** 2026-03-11

**Authors:** Julie Rebejac, Soyon Hong

**Affiliations:** 1 UK Dementia Research Institute, University College London London United Kingdom

**Keywords:** microglia, microglia replacement, leukodystrophies, transplantation, Mouse

## Abstract

A new method enables engineered immune progenitor cells to replace microglia in mice and reveals how a genetic mutation can lead to inflammation in the brain.

**Related research article** Nemec KM, Genevieve Uy VSC, Purnell FS, Elfayoumi B, Byerly L, O’Reilly ML, O’Brien CA, Aisenberg WH, Lombroso SI, Guo X, Blank N, Oon CH, Yaqoob F, Temsamrit B, Rawat P, Thaiss CA, Bailis W, Williamson AP, Wang Q, Bennett ML, Bennett FC. 2026. Microglia replacement by ER-Hoxb8 conditionally immortalized macrophages provides insight into Aicardi–Goutières Syndrome neuropathology. *eLife* **14**:RP102900. doi: 10.7554/eLife.102900.

The brain is the most complex organ in the human body and is estimated to contain over 3,000 cell types, including its resident immune cells, the microglia. Microglia are macrophages that colonise the central nervous system during embryonic development and persist throughout life (Ginhoux et al., 2010). Unlike most immune cells, they are not replenished from the bloodstream; instead, they maintain their population through local self-renewal.

Microglia are essential for brain function and homeostasis. During development, they preserve tissue integrity and refine neural circuits by eliminating weak synapses (Lawrence et al., 2024; Schafer et al., 2012). In adults, they continuously survey their environment, respond to neuronal activity, and contribute to synaptic plasticity (Badimon et al., 2020). However, during aging or disease, microglia can become dysregulated, leading to synaptic loss and neuroinflammation (Hong et al., 2016). Distinguishing between these protective and pathogenic states is therefore critical for understanding brain health and disease.

In some neurological disorders, the distinction between these two states is clear. Loss-of-function mutations impair microglial responses to damage (e.g. the gene *Trem2,* Wang et al., 2022), whereas other mutations can induce a toxic accumulation of lipids that ultimately leads to neuronal loss (e.g. *Hexb* in Sandhoff disease; Mader et al., 2025). These findings have prompted interest in strategies to replace native microglia with engineered cells for research and therapeutic studies. However, technical limitations have hindered progress as primary microglia are difficult to manipulate outside the body, and other cell alternatives, such as induced pluripotent stem cells, require substantial financial investment.

Now, in eLife, Mariko Bennett, Chris Bennett and colleagues – including Kelsey Nemec as first author – report a novel replacement strategy to overcome these obstacles and create alternative microglia surrogates (Figure 1; Nemec et al., 2026). The researchers used a method known as the estrogen-regulated homeobox B8 (Hoxb8) system to generate unlimited numbers of hematopoietic progenitors; these are immature cells from the bone marrow that are capable of differentiating into multiple lineages. Overexpressing Hoxb8 by adding estrogen maintained the cells in an undifferentiated and expandable state, whereas removing estrogen induced differentiation into macrophages.

**Figure 1.**
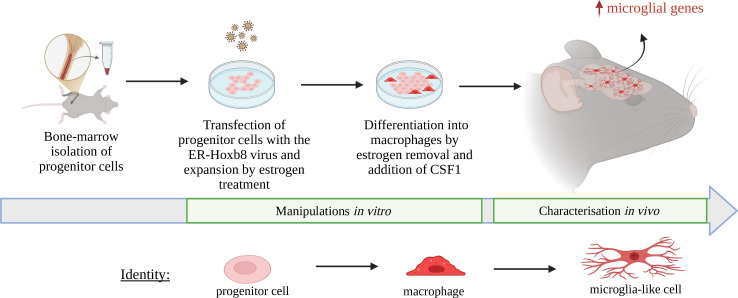
Microglia replacement strategy using genetically modified immature progenitor cells. Progenitor cells (pink) can be isolated from the bone marrow of mice (left) and genetically modified in vitro by adding the Estrogen-regulated-Hoxb8 virus (brown) to obtain an immature state where they continuously expand as progenitors (second from left). When estrogen is removed from the culture medium and CSF1 (growth factor) is added (second from right), the progenitor cells differentiate to become macrophages (red). When transplanted into mice (right), these macrophages turn into microglia-like cells (red branched shapes), allowing researchers to study how genes in microglia can alter the brain environment. Created in BioRender.com.

After transplantation into mice, the macrophages dispersed throughout the brain and adopted microglia-like features. They expressed genetic markers typical for microglia, including genes known to be involved in microglial function – Tmem119, P2yr12, and Hexb. However, the macrophages lacked Sall1, a transcription factor critical for establishing microglial identity (Buttgereit et al., 2016). These findings indicate that the engineered cells approximate – but do not fully recapitulate – the molecular identity of microglia found in the brain. Nevertheless, this approach would be faster, more cost-effective, and more experimentally tractable than current replacement strategies.

Nemec et al. leveraged this method to model Aicardi-Goutières syndrome, a rare genetic disorder caused by mutations in genes required for nucleic acid sensing and type I interferon production. Excessive interferon signalling in the brain drives pathological neuroinflammation, and microglia are thought to play an essential role as disease mediators (Hofer et al., 2024).

To model this condition, Nemec et al. genetically engineered cells to lack ADAR1, a gene often mutated in patients with Aicardi-Goutières syndrome. Without this gene, which helps prevent aberrant activation of innate immune pathways, most cells failed to differentiate into mature macrophages. Those that did differentiate were highly inflammatory, characterised by elevated interferon-stimulated gene expression and heightened immune activation. Transplantation of these cells into mice proved largely lethal, and the few surviving animals showed poor microglial engraftment. In contrast, progenitor cells carrying a patient-derived mutation in *Adar1* that only impairs the catalytic domain of this protein could be transplanted efficiently into mice and induced the production of an interferon-stimulated gene. These findings suggest that mutations in microglia are sufficient to initiate neuroinflammation in mice.

The approach used by Nemec et al. to replace microglia offers several experimental advantages. The progenitor cells used are amenable to genetic manipulation before differentiation and transplantation, and the timing of maturation can be precisely controlled. This flexibility makes it possible to investigate how specific mutations influence microglial function and brain health.

Beyond its research applications, this strategy offers an exciting avenue for microglial replacement as a potential therapy. Previous studies in mouse models of other genetic disorders suggest that microglial engraftment can improve neurological outcomes (Mader et al., 2025). Yet translation to humans faces major challenges. Current protocols rely on microglia-depleted or immunodeficient mice, conditions difficult to replicate safely. In addition, transplanted cells may never fully match primary microglia. It remains to be seen how closely replacement cells must mirror native microglia to be safe, if transplantation has any long-term effects on neural circuits and behaviour, and if similar findings can be seen using human models of microglia (Mancuso et al., 2019; Weerakkody et al., 2025).

Despite these uncertainties, Nemec et al. – who are based at the University of Pennsylvania, the Children’s Hospital of Philadelphia, and other institutions in the United States – provide a versatile tool for dissecting microglial biology and modelling neuroinflammatory diseases. By enabling controlled replacement of immune cells in the brain, this approach opens new avenues for understanding the role of microglia and exploring potential therapeutic interventions.
